# Supplementary material for: Organic Electrochemical Transistor with MoS2 Nanosheets Modified Gate Electrode for Sensitive Glucose Sensing
Source: Sensors (Basel). 2023 Aug 27;23(17):7449. doi: 10.3390/s23177449 (PMC10490818; doi:10.3390/s23177449)
Supplement: Supplementary file 1 [file sensors-23-07449-s001.zip › sensors-2554179-supplementary.pdf]

Supplementary Materials

Organic Electrochemical Transistor with MoS_2 Nanosheets Modified Gate Electrode for Sensitive Glucose Sensing

Jin Hu, Jiajia Dai, Caiping Huang, Xierong Zeng, Weiwei Wei, Zhezhe Wang, Peng Lin

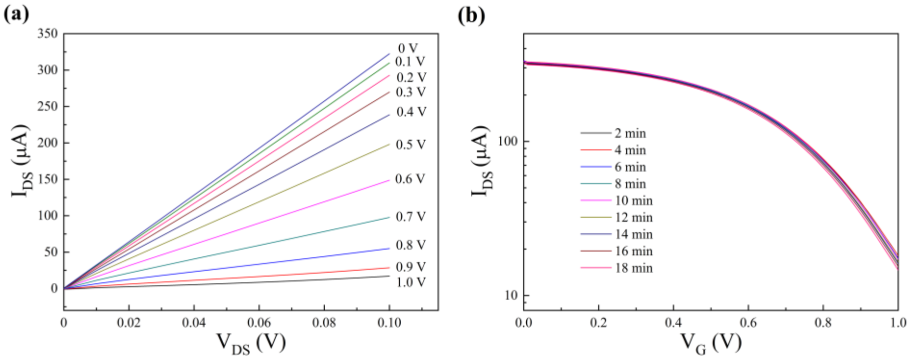

Figure S1. Characterization of OECT with a pure Pt gate electrode measured in PBS solution. (a) Output characteristic of the OECT. (b) Transfer characteristic of the OECT device at V_{DS} = 0.1 V.

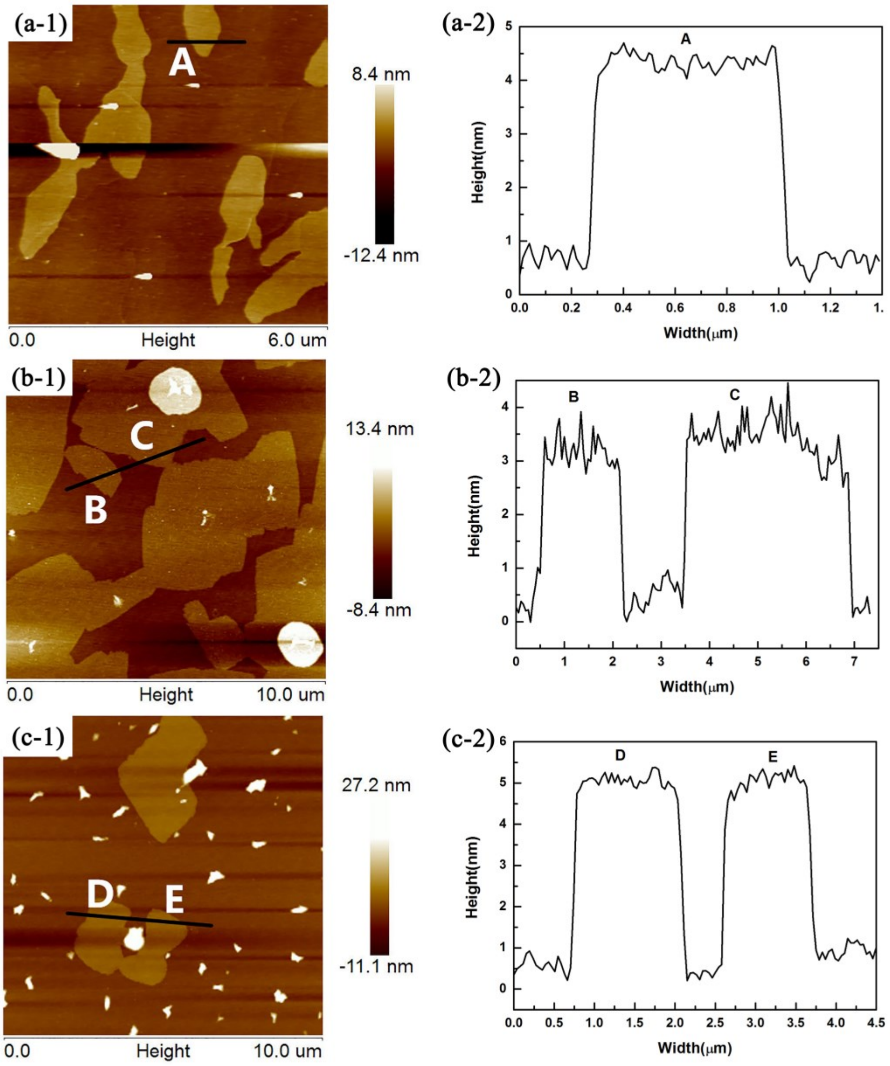

Figure S2. AFM images of MoS_2 nanosheets stripped with (a-1) NMP solvent, (b-1) sodium cholate solution, (c-1) aqueous alcohol. (a-2), (b-2), (c-2) are thickness of position A, B, C, D, E in AFM images of (a-1), (b-1), (c-1), respectively.
